# Draft Genome Sequence of a Haemophilus parainfluenzae Strain Isolated from a Patient with Chronic Obstructive Pulmonary Disease

**DOI:** 10.1128/MRA.00079-20

**Published:** 2020-03-26

**Authors:** Ad C. Fluit, Miquel B. Ekkelenkamp, Michael M. Tunney, J. Stuart Elborn, Malbert R. C. Rogers, Jumamurat R. Bayjanov

**Affiliations:** a Department of Medical Microbiology, University Medical Center Utrecht, Utrecht, The Netherlands; b School of Pharmacy, Queen’s University Belfast, Belfast, United Kingdom; c School of Medicine, Dentistry, and Biomedical Sciences, Queen’s University Belfast, Belfast, United Kingdom; Loyola University Chicago

## Abstract

Haemophilus parainfluenzae is considered part of the normal oropharyngeal flora but is known to occasionally cause infections. It is closely related to Haemophilus influenzae. Here, we report the genome sequence of H. parainfluenzae COPD-014-E1 O, which was cultured from the sputum of a patient with chronic obstructive pulmonary disease.

## ANNOUNCEMENT

Haemophilus parainfluenzae is a Gram-negative coccobacillus that is part of the normal oropharyngeal flora but has also been reported to cause endocarditis and sepsis (1, 2). It has been recovered from induced sputum samples from people with chronic obstructive pulmonary disease (COPD) and cystic fibrosis, but its role is still unclear (3–5). Here, we report the whole-genome sequence of H. parainfluenzae strain COPD-014-E1 O. The strain was cultured for 40 to 48 h on BCA medium (chocolate blood agar containing bacitracin) at 35°C to 37°C, in 5% CO_2_, from a sputum sample collected from a COPD patient in Belfast, Northern Ireland, United Kingdom. The H. parainfluenzae isolate was collected in a previous study with ethical approval from the Office for Research Ethics Northern Ireland; patients in the study consented for collected samples to be stored indefinitely to facilitate future research.

Bacterial DNA was isolated with the QIAcube DNeasy blood and tissue kit with the protocol for bacterial or yeast DNA with enzymatic lysis (Qiagen, Germany) after pretreatment with 3 μg/ml lysozyme for 30 min at 37°C. A DNA library was prepared using the Nextera XT kit and protocol (Illumina, CA) and was subsequently sequenced on an Illumina NextSeq platform using the 2 × 150-bp sequencing kit. All reads were trimmed with seqtk trimfq version 1.3 (https://github.com/lh3/seqtk), with an error rate threshold of 0.001, and Nextera transposase sequences were removed with Trim Galore version 0.5.0 (https://www.bioinformatics.babraham.ac.uk/projects/trim_galore). Contigs were assembled with SPAdes genome assembler version 3.6.2, and contigs larger than 500 bp, with at least 10× coverage, were further analyzed. The total number of paired-end reads was 7,039,708. Assembly resulted in 187 contigs with a total length of 2,127,067, an average coverage of 439×, and a GC content of 38.9%. This finding compares to a length range of 1.9 to 2.1 Mb for this species. The assembled contigs were annotated by Rapid Annotation using Subsystems Technology (RAST) (6). Further analysis for the presence of resistance genes was performed with ResFinder version 3.1 (DTU, Denmark) (7). Default software settings were used except the following settings for SPAdes: only-assembler, careful, k 57,97,127, cov-cutoff 10.

Gene annotation using RAST identified 2,099 coding sequences and 51 RNAs. Using both RAST and ResFinder, no acquired antibiotic resistance genes were found in this strain. No mobile elements were detected, with the exception of a putative prophage. Further analysis showed that 3 contigs (approximately 22, 8, and 7 kb) harbored only phage-related genes and genes encoding hypothetical proteins. Three additional contigs encoded between 1 and 3 phage proteins at one of the ends. Genes encoding a putative integrase, capsid and tail proteins, prohead protease, portal protein, head-tail adaptor, terminase, shock protein E, replication protein, nuclease, and repressor were also identified. Two putative phage integrases were identified, suggesting that the strain may carry two bacteriophages; however, integrases from different mobile elements are sometimes highly similar, making accurate prediction difficult.

### Data availability.

This whole-genome shotgun project has been deposited under BioProject PRJNA562164 (BioSample SAMN12635205). The reads can be accessed under SRA accession number SRR10875778. This whole-genome shotgun project has been deposited at DDBJ/ENA/GenBank under accession number VTSN00000000, and the nonannotated genome assembly is available under accession number GCA_009914785. The version described in this paper is version VTSN01000000.
